# Pedicle screw fixation and posterior fusion for lumbar degenerative diseases: effects on individual paraspinal muscles and lower back pain; a single-center, prospective study

**DOI:** 10.1186/s12891-016-0927-9

**Published:** 2016-02-06

**Authors:** Jae-Ryong Cha, Yong-Chan Kim, Chulyoung Jang, Woo-Kyoung Yoo, Ji Hao Cui

**Affiliations:** Department of Orthopedic Surgery, Ulsan University Hospital, College of Medicine, Ulsan University, 290-3 Cheonha-Dong, Ulsan-si, Dong-Ku South Korea; Department of Orthopaedic Surgery, Hallym University Sacred Heart Hospital, Hallym University, 896 Pyeongchon-dong, Dongan-gu, Anyang-si South Korea; Department of Physical Medicine and Rehabilitation, Hallym University Sacred Heart Hospital, College of Medicine, Hallym University, 896 Pyeongchon-dong, Dongan-gu, Anyang-si South Korea; Department of Orthopaedic Surgery, The Fourth Affiliated Hospital of Guangzhou Medical University, 195 Dongfeng Xi Road, Guangshou, China

**Keywords:** Pedicle screw fixation, Posterior fusion, Paraspinal muscle, Reinnervation, Lower back pain

## Abstract

**Background:**

To the best of our knowledge, there have been no reports on the points at which the denervated multifidus and erector spinae muscles become reinnervated after pedicle screw fixation and posterior fusion in patients with lumbar degenerative diseases. Our study was designed to confirm reinnervation of denervated paraspinal muscles following pedicle screw fixation and posterior fusion and to confirm alleviation of the patients’ lower back pain (LBP).

**Methods:**

In this prospective study, we enrolled 67 patients who had undergone pedicle screw fixation and posterior fusion. The surgery had alleviated their leg pain, but the patients complained of LBP at the L3-5 level 3 months after the surgery. The patients were divided into two groups (I and II) according to the level at which pain was experienced. Paraspinal mapping scores were recorded preoperatively and 3, 6, 12, and 18 months postoperatively. Oswestry Disability Index and visual analogue scale scores were determined. Regression analyses using a general linear model and a mixed model were performed.

**Results:**

Pedicle screw fixation and posterior fusion significantly denervated the multifidus and erector spinae not only in the surgical segment, but also in adjacent segments. Group I patients displayed reinnervation in the denervated erector spinae and multifidus muscles at 12 and 18 months, respectively. In contrast, group II showed reinnervation only in of the denervated erector spinae of the upper segment at 18 months, with no other areas of reinnervation. Postoperative LBP was significantly diminished at 12 months in group I and at 18 months in group II. There was also significantly less LBP at 6 months (prior to reinnervation of the paraspinal muscles).

**Conclusions:**

The denervated multifidus and erector spinae muscles at L4–5, which had been denervated using pedicle screw fixation and posterior fusion, were significantly reinnervated at 18 months postoperatively, whereas patients with denervation at L3–5 had only a tendency to be reinnervated at follow-up. Postoperative LBP in these patients was significantly diminished at the follow-up visits.

## Background

Posterior spinal fusion, followed by instrumented spinal fusion, is the main strategy for treating degenerative lumbar disease (DLD). It is associated, however, with postoperative complications, such as lower back pain (LBP), which have been described in the literature [1–3]. Intraoperatively, muscle dissection from the vertebral processes and prolonged retraction cause ischemia and denervation of the paraspinal muscles, resulting in degenerative changes in these muscles and pain [4]. It has been reported that patients with chronic or postoperative LBP have less well developed paraspinal muscles than those in age-matched, normal, healthy individuals [5]. It has also been reported that there is a postoperative reduction in the cross-sectional area of paraspinal muscles, such as the multifidus [6]. One previous study showed atrophy of type II fibers and internal structural changes in type I fibers of multifidus muscles in these patients [7]. To date, there has been a persistent interest in postoperative changes in paraspinal muscles, including their decreased thickness on ultrasonography [8], edema and fat degeneration on magnetic resonance imaging (MRI) [9], and low myogenic potential on neurophysiological tests [10]. Others have reported that paraspinal muscles are vulnerable to denervation and atrophy because of their dissection and retraction and the immobilized spinal segment due to fusion during posterior spinal operations [11].

It remains uncertain, however, whether damaged paraspinal muscles can recover after posterior spinal surgery. In addition, there are no reports regarding the neurophysiology of paraspinal muscles at the surgical and adjacent levels.

Given this background, we set out to confirm (or deny) reinnervation of denervated paraspinal muscles after pedicle screw fixation and posterior fusion. We also wanted to compare the timing of reinnervation between the denervated multifidus and erector spinae depending on the surgical level in patients with a DLD. Finally, we analyzed the relation between the point at which postoperative LBP was alleviated and the point at which the denervated multifidus and erector spinae began reinnervation.

## Methods

### Study population and design

This study was approved by Forum for Ethical Review Committees in Asia & the Western Pacific (FERCAP). A written informed consent was obtained from all participants in this study. We enrolled a total of 67 patients whose leg pain had been alleviated by pedicle screw fixation and posterior fusion at the L3-5 level at our institution between July 2009 and November 2012 but who now, 3 months later, complained of LBP. The patients were followed up for a minimum of 18 months. This clinical series of patients comprised 15 men and 52 women (mean age 62.2 years, range 41–82 years; mean body mass index 24.4 kg/m^2^, range 19.4–33.5 kg/m^2^). Their underlying diseases included 54 cases of spinal stenosis and 13 cases of spondylolisthesis.

The patients were divided into two groups depending on the surgical level: group I (36 patients), who had undergone pedicle screw fixation and posterior fusion after total facetectomy at the L4–5 level; and group II (31 patients), who had undergone pedicle screw fixation and posterior fusion after total facetectomy at the L3–5 level. All surgical dissections were performed via a midline posterior approach, with the same retractor used to expose the surgical field.

The study was designed to analyze paraspinal muscles (e.g., multifidus and erector spinae) at three spinal levels: the surgical segment (L4–5 in both groups), upper segment (L3–4 in group I and L2–3 in group II), and lower segment (L5–S1 in both groups). To evaluate changes in paraspinal muscles at 3, 6, 12, and 18 months postoperatively—compared with that preoperatively—electromyography (EMG) was performed according to the guidelines of the American Association of Electrodiagnostic Medicine using the NeuroScreen (Jaeger-Toennies, Würzburg, Germany). To quantify any abnormal spontaneous activity, we performed paraspinal mapping. In addition, we undertook simplified mini-paraspinal mapping [12]. The positive sharp wave, fibrillation potential, complex repetitive discharge, and fasciculation potential were recorded on both sides and then averaged. Measurements were evaluated based on five grades (0, 1+, 2+, 3+, 4+) (Table 1). Thus, we compared the degree of denervation of the multifidus and erector spinae muscles at 3, 6, 12, and 18 months postoperatively compared with that observed preoperatively.

**Table 1:** Paraspinal mapping scoring system for points of insertion

Score	Criteria
0	No reliable data obtained^a^
−	No reproducible spontaneous activity^b^
+	A single, reproducible train of fibrillation potentials
++	More than one train of fibrillation potentials
+++	Numerous fibrillation potentials at more than one depth
++++	Fibrillation potentials fill the screen

^a^Two or more motor units are interfering with inspection for possible waves, the insertion contracting the bone before contracting muscle, or adipose depth being greater than needle length

^b^No consideration for spontaneous activity in association with periosteum or motor end-plate noise

**Fig. 1:**
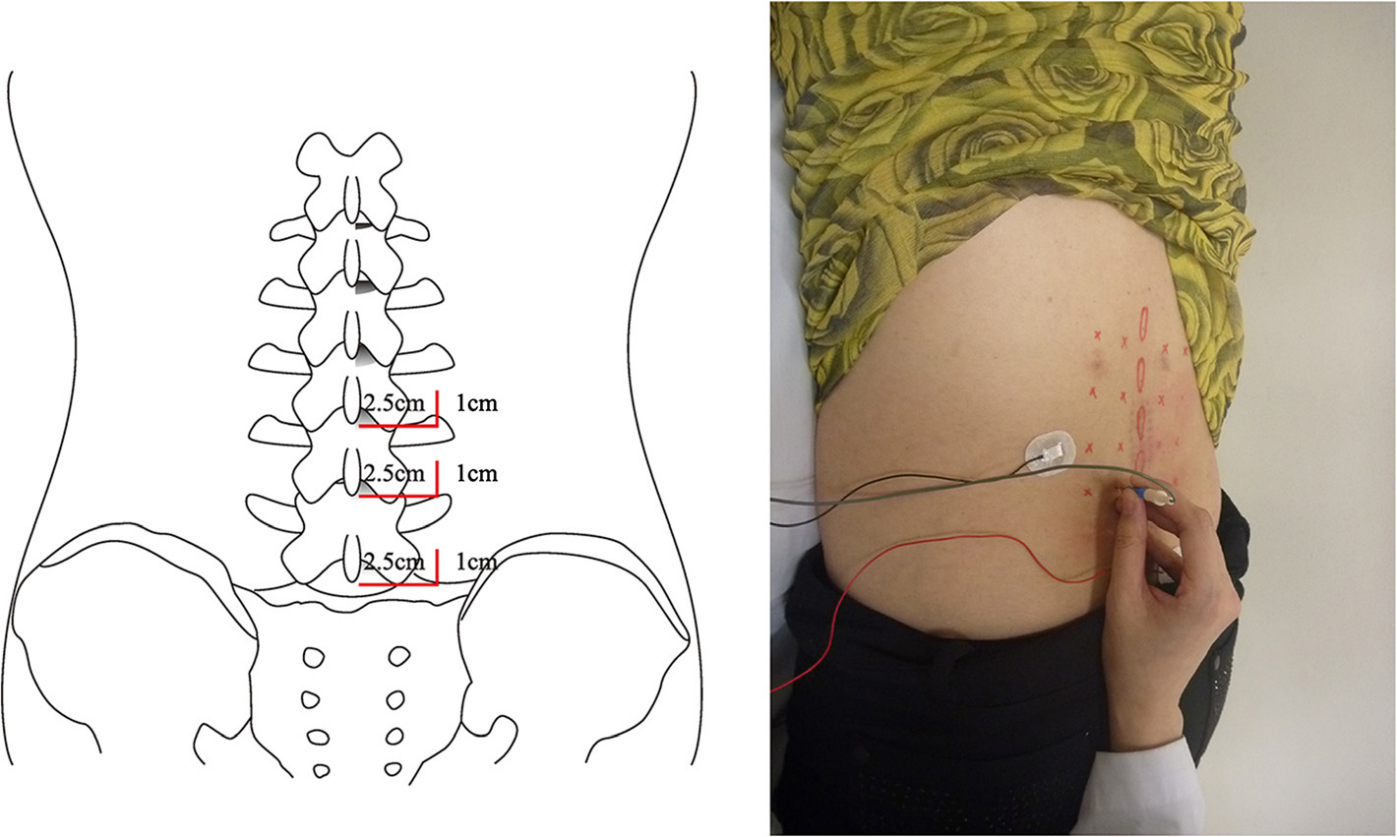
Placement of the needle from the surgical level to adjacent segments from the posterior view of the lumbar spine

To assess the multifidus at each segment, the sites of needle insertion were marked in the region 2.5 cm lateral and 1 cm cranial to the inferior border of the spinous process of the lower lumbar interbody (Fig. 1). As a landmark for surgical removal during the decompression procedure at the L4 spinous process, we set the midpoint between the spinous process of L3 and L5. When the depth of insertion reached 40 mm, we considered that the needles were inserted into the multifidus muscle. (Severely obese patients were excluded from the current study.) We then, again, placed the needle in the cranial direction at an angle of 45° and in the caudal direction at an angle of 45° toward the midline. To ensure access to the erector spinae, we placed the needle in the cranial direction at an angle of 45°and in the caudal direction at an angle of 45° from the midline, as previously. We then confirmed these landmarks on ultrasonography.

We also evaluated clinical outcomes, except for leg pain, based solely on the LBP, using both the visual analogue scale (VAS) and the Oswestry Disability Index (ODI) preoperatively and at 3, 6, 12, and 18 months postoperatively. We did not use the ODI to evaluate the patients’ sexual activity.

### Statistical analysis

Statistical analysis was done using SPSS version 10.0 for Windows software (SPSS Inc., Chicago, IL, USA). For statistical analysis, we performed a regression analysis of descriptive variables and a mixed model analysis preoperatively and at 3, 6, 12, and 18 months postoperatively. We also performed a Mann − Whitney test to analyze differences in variables between the upper, surgical, and lower segments. A value of *P* < 0.05 was considered to indicate statistical significance.

## Results

### Changes in the multifidus muscle on EMG

**Fig. 2:**
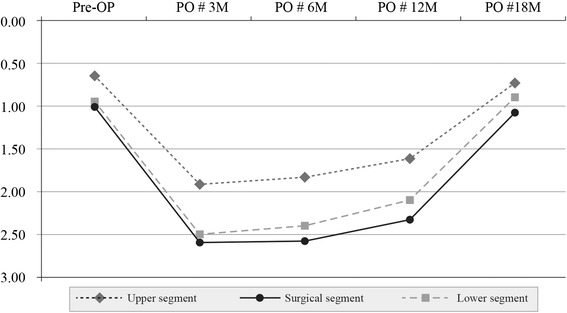
Changes in the multifidus muscle in group I. Pre-OP: preoperative; PO # 3 M, 6 M, 12,M, 18 M: 3, 6, 12, 18 months postoperatively

In group I, denervation in the upper segment of the multifidus muscle was 0.66+ preoperatively, 1.91+ at 3 months, 1.83+ at 6 months, 1.62+ at 12 months, and 0.72+ at 18 months. In the multifidus surgical segment, they were, respectively, 1.01+, 2.59+, 2.58+, 2.31+, and 1.08+. The corresponding values in the multifidus lower segment were 0.93+, 2.50+, 2.40+, 2.11+, and 0.89+, respectively. These results indicate that there was reinnervation of the denervated multifidus at the surgical and adjacent segments at 18 months in group I (Table 2) (Fig. 2).

**Table 2:** Paraspinal mapping scores for group I

Segment	Preop	PO#3 M (*P* value)	PO#6 M (*P* value)	PO#12 M (*P* value)	PO#18 M (*P* value)
Multifidus muscle					
Upper	0.66 ± 0.60	1.91 ± 0.88 (<0.0001)	1.83 ± 1.01 (<0.0001)	1.62 ± 1.11 (<0.0001)	0.72 ± 0.49 (0.5324)
Surgical	1.01 ± 0.61	2.59 ± 1.03 (<0.0001)	2.58 ± 1.19 (<0.0001)	2.31 ± 1.01 (<0.0001)	1.08 ± 0.62 (0.5824)
Lower	0.93 ± 0.74	2.50 ± 0.89 (<0.0001)	2.40 ± 1.05 (<0.0001)	2.11 ± 1.19 (<0.0001)	0.89 ± 0.64 (0.4251)
Erector spinae muscle					
Upper	0.64 ± 0.61	1.75 ± 0.78 (<0.0001)	1.58 ± 0.75 (<0.0001)	0.70 ± 0.52 (0.5187)	0.67 ± 0.59 (0.8027)
Surgical	1.05 ± 0.55	2.25 ± 1.01 (<0.0001)	2.05 ± 0.98 (<0.0001)	1.23 ± 0.60 (0.2155)	0.99 ± 0.65 (0.6412)
Lower	0.82 ± 0.71	2.20 ± 0.84 (<0.0001)	1.92 ± 0.80 (<0.0001)	1.02 ± 0.81 (0.6029)	0.85 ± 0.64 (0.8684)

Statistical significance at *P* < 0.05

The upper segment indicates L3–4 in group I and L2–3 in group II; surgical segment indicates L4–5; lower segment, L5–S1, respectively

Preop and POM 3, 6, 12, and 18 indicate “preoperatively” and “at 3, 6, 12, and 18 months, postoperatively,” respectively

**Fig. 3:**
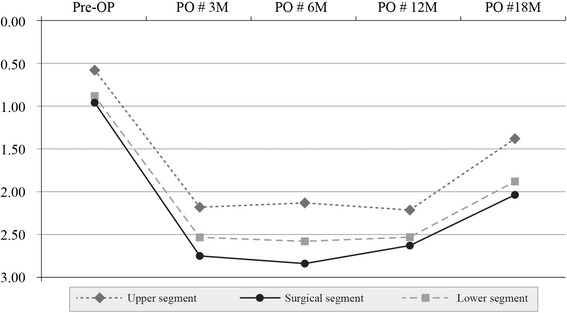
Changes in the multifidus muscle in group II. Pre-OP: preoperative; PO # 3 M, 6 M, 12,M, 18 M: 3, 6, 12, 18 months postoperatively

In group II, denervation in the multifidus muscle at the upper segment was 0.58+ preoperatively, 2.19+ at 3 months, 2.14+ at 6 months, 2.22+ at 12 months, and 1.37+ at 18 months. The corresponding values in the multifidus surgical segment were 0.96+, 2.75+, 2.83+, 2.63+, and 2.03+, respectively. In the multifidus lower segment, they were 0.89+, 2.53+, 2.58+, 2.53+, and 1.87+, respectively. These results indicate that there was no reinnervation of the denervated multifidus in the surgical and adjacent segments at 18 months in group II (Table 3) (Fig. 3).

**Table 3:** Paraspinal mapping scores for group II

Segment	Preop	PO#3 M (*P* value)	PO#6 M (*P* value)	PO#12 M (*P* value)	PO#18 M (*P* value)
Multifidus muscle					
Upper	0.58 ± 0.81	2.19 ± 1.04 (<0.0001)	2.14 ± 0.87 (<0.0001)	2.22 ± 0.96 (<0.0001)	1.37 ± 0.88 (0.0352)
Surgical	0.96 ± 0.81	2.75 ± 0.71 (<0.0001)	2.83 ± 0.69 (<0.0001)	2.63 ± 0.98 (<0.0001)	2.03 ± 0.82 (<0.0001)
Lower	0.89 ± 0.85	2.53 ± 1.03 (<0.0001)	2.58 ± 0.97 (<0.0001)	2.53 ± 1.05 (<0.0001)	1.87 ± 0.74 (<0.0001)
Erector spinae muscle					
Upper	0.79 ± 0.70	1.94 ± 0.95 (<0.0001)	1.81 ± 0.79 (<0.0001)	1.72 ± 1.16 (0.0012)	1.06 ± 0.41 (0.0895)
Surgical	0.85 ± 0.66	2.57 ± 0.73 (<0.0001)	2.58 ± 0.75 (<0.0001)	2.42 ± 0.81 (<0.0001)	1.49 ± 0.60 (0.0312)
Lower	0.83 ± 0.70	2.33 ± 0.83 (<0.0001)	2.51 ± 1.01 (<0.0001)	2.33 ± 0.93 (<0.0001)	1.42 ± 0.42 (0.0122)

Statistical significance at *P* < 0.05

The upper segment indicates L3–4 in group I and L2–3 in group II; surgical segment indicates L4–5; lower segment, L5–S1, respectively

Preop and POM 3, 6, 12, and 18 indicate “preoperatively” and “at 3, 6, 12, and 18 months, postoperatively,” respectively

### Changes in the erector spinae on EMG

**Fig. 4:**
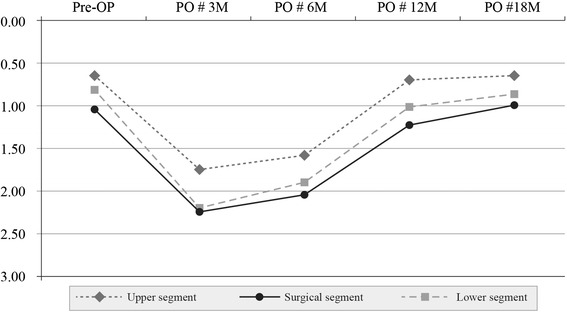
Changes in the erector spinae muscle in group I. Pre-OP: preoperative; PO # 3 M, 6 M, 12,M, 18 M: 3, 6, 12, 18 months postoperatively

In group I, denervation of the erector spinae upper segment was 0.64+ preoperatively, 1.75+ at 3 months, 1.58+ at 6 months, 0.70+ at 12 months, and 0.67+ at 18 months. The corresponding values in the surgical segment were 1.05+, 2.25+, 2.05+, 1.23+, and 0.99+, respectively. The values in the lower segment were 0.82+, 2.20+, 1.92+, 1.02+, and 0.85+, respectively. These results indicate that, in group I, there was reinnervation of the denervated erector spinae in the surgical and adjacent segments at 12 months (Table 2) (Fig. 4).

**Fig. 5:**
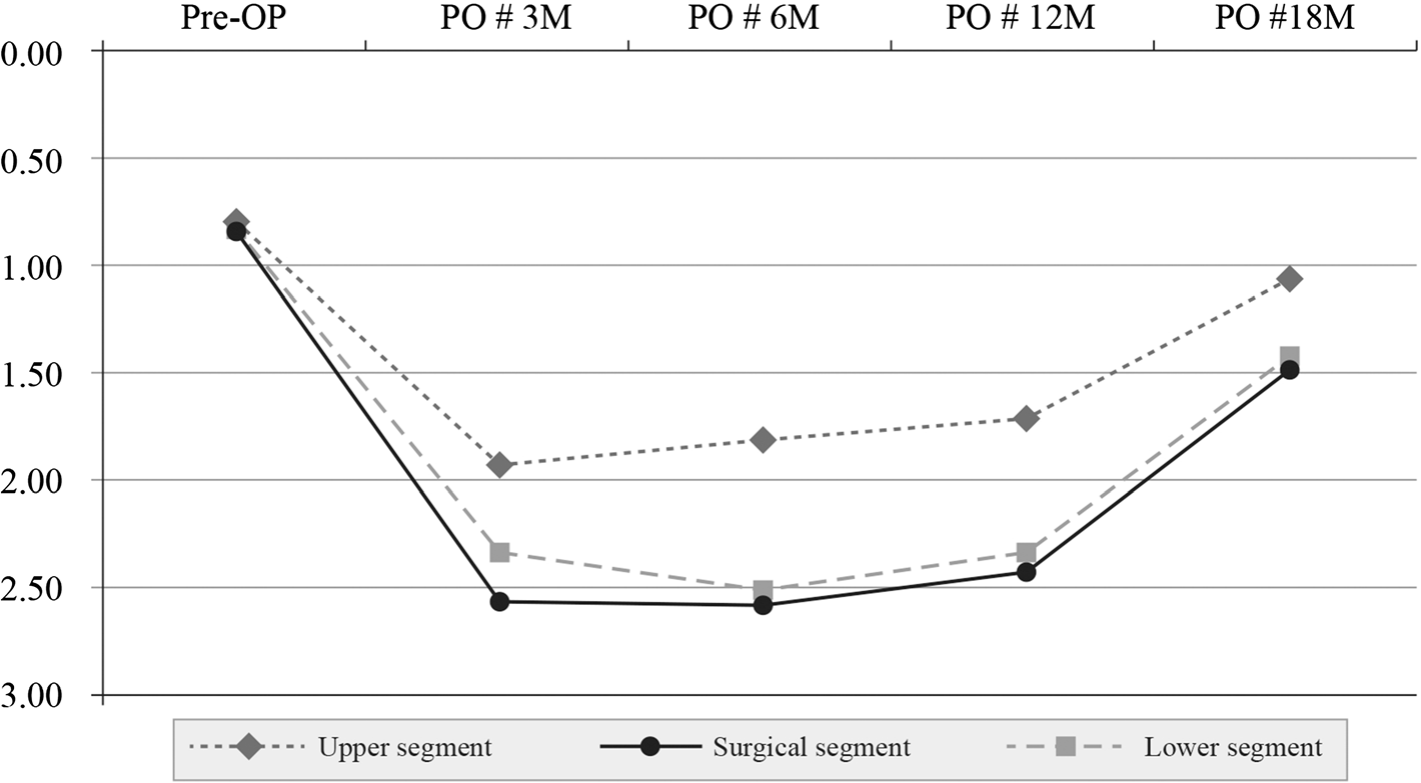
Changes in the erector spinae muscle in group II. Pre-OP: preoperative; PO # 3 M, 6 M, 12,M, 18 M: 3, 6, 12, 18 months postoperatively

In group II, denervation of the erector spinae in the upper segment was 0.79+ preoperatively, 1.94+ at 3 months, 1.81+ at 6 months, 1.72+ at 12 months, and 1.06+ at 18 months. In the surgical segment, the corresponding values were 0.85, 2.57+, 2.58+, 2.42+, and 1.49+, respectively. The values in the lower segment were 0.83+, 2.33+, 2.51+, 2.33+, and 1.42+, respectively. These results indicate that, in group II, there was reinnervation only in the denervated erector spinae of the upper segment at 18 months (Table 3) (Fig. 5).

### Changes in LBP

In group I, the mean VAS/ODI scores were 4.49/15.14 preoperatively, 6.95/25.42 at 3 months, 4.77/14.29 at 6 months, 1.21/4.99 at 12 months, and 0.67/1.89 at 18 months. In group II, these values were 4.11/14.06, 7.28/28.00, 6.39/24.17, 5.61/19.28, and 3.44/9.17, respectively (Table 4). These results indicate that there was significant aggravation of LBP in both groups immediately postoperatively. However, there was also significant alleviation of LBP at 12 months in group I and at 18 months in group II. In addition, there was significantly less LBP at 6 months (prior to reinnervation) in paraspinal muscles.

**Table 4:** Changes in lower back pain for each group: significance (P)

Parameter	Preop	PO#3 M	PO#6 M	PO#12 M	PO#18 M
Group I					
VAS	4.49 ± 0.65	6.95 ± 0.84	4.77 ± 1.12	1.21 ± 0.66	0.67 ± 0.54
Preop vs PO		<0.0001	0.0620	<0.0001	<0.0001
Previous PO vs ultimate PO			0.0047	<0.0001	0.0325
ODI	15.14 ± 3.93	25.42 ± 3.47	14.29 ± 4.25	4.99 ± 2.33	1.89 ± 0.48
Preop vs PO		<0.0001	0.7530	<0.0001	<0.0001
Previous PO vs ultimate PO			<0.0001	<0.0001	<0.0001
Group II					
VAS	4.11 ± 0.90	7.28 ± 0.75	6.39 ± 1.38	5.61 ± 1.65	3.44 ± 1.69
Preop vs PO		<0.0001	0.0110	0.0390	0.0414
Previous PO vs ultimate PO			0.5540	0.8750	0.0120
ODI	14.06 ± 2.58	28.00 ± 4.78	24.17 ± 4.64	19.28 ± 3.79	9.17 ± 3.76
Preop vs PO		<0.0001	<0.0001	0.0012	<0.0001
Previous PO vs ultimate PO			0.0310	0.0210	<0.0001

Preop and POM 3, 6, 12, and 18 indicate “preoperatively” and “at 3, 6, 12, and 18 months, postoperatively,” respectively. *PO* indicates postoperative, *VAS* visual analogue scale, *ODI* Oswestry Disability Index

### Changes in paraspinal muscles in adjacent segments

In group I, there was a significant difference in the degree of changes in multifidus and erector spinae muscles between the upper and lower segments at 3, 6, and 12 months postoperatively (*P* < 0.05). There were no such differences at 18 months, however (*P* = 0.0923 and *P* = 0.1042, respectively) (Figs. 2, 4).

In group II, there was a significant difference in the degree of changes in the multifidus and erector spinae muscles between the upper and lower segments at 3, 6, 12, and 18 months postoperatively (*P* < 0.05) (Figs. 3, 5). It is of note that there was slight damage to both the multifidus and erector spinae muscles in the upper segment during surgery. Reinnervation occurred earlier in the upper segments than in the lower segments.

## Discussion

It is widely known that patients undergoing lumbar surgery are at increased risk of developing atrophy of the lumbar extensors [13]. Yong et al., who conducted an animal study to compare a fusion group and a control group, reported that there was a significant decrease in the root mean square (RMS) and median frequency (MF)—which served as indicators of the activity of paraspinal muscles—in the fusion group at the 6-month follow-up when compared with the preoperative values. These authors also showed that histologically there was also a significant decrease in the volume of muscle fibers, which served as an indicator of amyotrophy [14]. A more recent human study observed significantly increased denervation at the 6-month follow-up [15]. Our study showed similar results in that both multifidus and erector spinae muscles were still significantly denervated at the 6-month follow-up (compared with their preoperative status). In terms of the changes in adjacent levels, Yong et al. reported that there was a significant increase in the RMS and MF of paraspinal muscles at the adjacent cranial and caudal levels when compared with those that had been subjected to surgery [14]. The current study, however, demonstrated that there was a significant difference in the degree of change in multifidus and erector spinae muscles between the adjacent cranial and caudal levels. We think it may result from the different subjects being studied (adult New Zealand white rabbits versus adult humans), the degree of damage to the paraspinal muscles, and the fusion level.

For the purpose of avoiding a bias when evaluating the degree of denervation in the paraspinal muscles on EMG, we limited the spinal levels to L3–5. There were significant degrees of denervation in the paraspinal muscles at the surgical level and its adjacent levels at 3 months. This might be due to the retraction, compression, and intraoperative damage to the posterior primary ramus and disuse muscle atrophy due to fusion and orthoses.

Waschke et al. demonstrated there was slightly decreased denervation at the 12-month follow-up in adult humans [15]. In the current study, we found that, in group I, there was more rapid reinnervation in the upper spinal segments that at other levels. This might be due to technical problems such as less traction, less severe direct damage to paraspinal muscles during dissection, and a smaller area of the multifidus being removed. We also assumed that a greater amount of paraspinal muscles’ dissection in the cranial and lateral directions and a more immobilized spinal segment might lead to slower reinnervation in group II than in group I.

In group II, although there was no significant reinnervation of the denervated multifidus and erector spinae muscles until the 18-month follow-up, the upper segment of the denervated erector spinae was significantly reinnervated at 18 months. Also, the other muscles of each segment had a tendency to be reinnervated during serial follow-up evaluations. These results suggest that the postoperative denervation in paraspinal muscles resulting from one- and two-level pedicle screw fixation and posterior fusion might not be a permanent phenomenon. Further studies are therefore warranted to confirm the significant reinnervation of paraspinal muscles following multiple levels of pedicle screw fixation and fusion.

In the current study, we assumed that the degrees of reinnervation in denervated erector spinae and multifidus muscles at the 12- and 18-month follow-up evaluations provide evidence that muscle function is likely to recover to the preoperative level even in patients undergoing an instrumentation and fusion procedure.

As described by Macintosh and Bogduc, there is limited evidence to explain the correlation between denervated paraspinal muscles and failed back surgery syndrome (FBSS) in patients undergoing posterior lumbar surgery [16]. In contrast, there are many reasons for an unsatisfactory clinical outcome after posterior instrumentation and fusion of the lumbar spine. Among them, dysfunction of the paraspinal muscles due to atrophy is one of the most discussed issues [17]. Sihvonen et al. reported that the degree of denervation in paraspinal muscles was significantly greater in patients with FBSS than in those with satisfactory surgical outcomes [6]. Ranaten et al. also suggested that inactivation of and damage to axons might be associated with atrophy of type 2 muscle fibers and unsatisfactory clinical outcomes [18]. Wilbourne and Aminoff demonstrated that denervation of paraspinal muscles leads to significant changes in spinal biomechanics, which could cause LBP [19]. In terms of the clinical consequence of denervation of paraspinal muscles, the current study showed results similar to those reported for previous studies.

Moreover, there was significant alleviation of LBP at the 12-month follow-up in group I and at the 18-month follow-up in group II. Thus, the LBP diminishes prior to reinnervation in denervated paraspinal muscles.

## Conclusions

The denervated multifidus and erector spinae accomplished by pedicle screw fixation and posterior fusion at L4–5 had significantly reinnervated at 18 months, and those at L3–5 had a tendency to be reinnervated at longer follow-up intervals. Postoperative LBP in these patients was significantly diminished at follow-up evaluations, but prior to reinnervation in denervated paraspinal muscles.

### Consent

A written informed consent was obtained from all the participants in this study.

## Abbrevations

DLD: Degenerative lumbar disease
LBP: Lower back pain
MRI: Magnetic resonance imaging
FERCAP: Forum for Ethical Review Committees in Asia & the Western Pacific
EMG: Electromyography
BMI: Body mass index
VAS: Visual Analogue Scale
ODI: Oswestry Disablity Index
RMS: Root mean square
MF: Median frequency
FBSS: Failed back surgery syndrome
